# An Open-Source Toolkit To Expand Bioinformatics Training in Infectious Diseases

**DOI:** 10.1128/mBio.01214-21

**Published:** 2021-07-06

**Authors:** Alexander S. F. Berry, Camila Farias Amorim, Corbett L. Berry, Camille M. Syrett, Elise D. English, Daniel P. Beiting

**Affiliations:** a Department of Pathobiology, University of Pennsylvania, Philadelphia, Pennsylvania, USA; b Department of Biomedical Sciences, School of Veterinary Medicine, University of Pennsylvania, Philadelphia, Pennsylvania, USA; c Division of Gastroenterology, Hepatology, and Nutrition, Children’s Hospital of Philadelphia, Philadelphia, Pennsylvania, USA; University of Arizona

**Keywords:** education, bioinformatics, genomics, infectious disease, transcription

## Abstract

As access to high-throughput sequencing technology has increased, the bottleneck in biomedical research has shifted from data generation to data analysis. Here, we describe a modular and extensible framework for didactic instruction in bioinformatics using publicly available RNA sequencing data sets from infectious disease studies, with a focus on host-parasite interactions. We highlight lessons learned from adapting this course for virtual learners during the coronavirus disease 2019 (COVID-19) pandemic.

## PERSPECTIVE

The demand is high for instructional resources that effectively engage traditional “bench” biologists in learning bioinformatics. Since genome-wide transcriptional profiling was first carried out in yeast over a decade ago (1), RNA sequencing (RNA-seq) has become a widely used tool for addressing many questions in studies of host-pathogen interactions (2–6). Unfortunately, in our experience, most didactic instruction for RNA-seq data analysis occurs in the context of general workshops or short courses that use toy data sets and that are often not structured in a way that affords sufficient time to teach best practices for coding. In 2015, we began a semester-long course with the goal of empowering students to take a “do-it-yourself” (DIY) approach to learning transcriptomics using the R programming environment and the Bioconductor suite of software packages. Over the past 6 years, we have refined this course to create a comprehensive, fully virtual, and open-source set of resources suitable for learners ranging from high school students to graduate students and postgraduate professionals. To facilitate broad access, all teaching materials are freely available at https://diytranscriptomics.com.

Several hardware and software developments make this an opportune time for curriculum development with RNA-seq data as the focal point: (i) the discontinuation of major sequencing platforms from Applied Biosystems (SOLiD) and Roche (454 pyrosequencing) beginning around 2013 left Illumina’s sequencing by synthesis the dominant technology, allowing instructors to focus on a single platform and nomenclature (7); (ii) the development of lightweight “pseudoalignment” algorithms allows read mapping to be carried out with modest computing resources, obviating the need to teach students how to communicate with a centralized computing cluster as a prerequisite for data analysis and allowing students to run alignments directly on their laptops, oftentimes in the span of a single class (8, 9); (iii) these efficient algorithms, together with troves of publicly available RNA-seq data, have catalyzed efforts that enable command-line access to data from hundreds of thousands of samples (10, 11); (iv) the community of computational biologists using R has grown tremendously, and its growth has engendered a rich and integrated user interface (12, 13); and (v) there have been major developments in easy-to-use interactive graphics, dynamic reports, and Web apps in the R environment, making it easy for students to turn static plots into dynamic data visualizations (14).

### Parasites are ideal tools for studying gene expression.

Analysis of host-parasite gene expression data provides an excellent opportunity to teach fundamental concepts in both parasitology and immunology. Parasite life cycles involve complex developmental transitions that coincide with remarkable alterations in gene expression, and new single-cell technologies enable high-resolution profiling of these life cycles. RNA-seq data sets from different developmental stages provide learners with insight into mechanisms of host cell invasion, immune evasion, parasite maturation, sexual differentiation, and reproduction. In addition, parasites trigger robust immune and tissue repair responses in their hosts, providing an opportunity to move beyond parasite biology to consider and discuss how pathogens elicit immune responses and what the consequences of these responses may be for the outcome of infection and the development of pathological responses. To take full advantage of this concept, our course includes data-driven virtual labs derived from real infectious disease studies. Each lab was designed to highlight fascinating and unique aspects of host-pathogen biology, including “just-in-time” gene expression during the erythrocytic cycle of Plasmodium falciparum, the helminth response to praziquantel treatment, the expression of microexon genes in Schistosoma mansoni, the activation of canonical antiviral responses by some intracellular protozoa, parasite strain-specific polarization of macrophages via Toxoplasma gondii secreted virulence factors, and immune activation during severe acute respiratory syndrome coronavirus 2 (SARS-CoV-2) infection.

### A modular approach to teaching coding.

**FIG 1:**
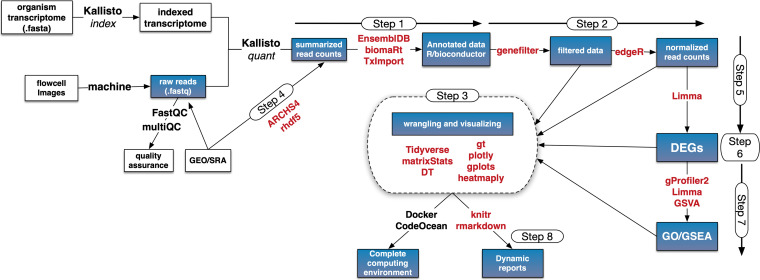
Complex workflows broken into modular “step” scripts. Learners progress through the course using a series of R step scripts. This process incrementally builds a computational workflow and culminates in learners producing an Rmarkdown report that summarizes all code and outputs from the course. Blue boxes indicate module topics covered in detail in the course. White boxes indicate topics discussed but not covered in detail. Red text denotes R packages used throughout the course, while black text denotes Web-based or command-line tools outside the R/Bioconductor environment.

The course is organized into 13 to 16 2-h modules, each of which includes lecture videos and slides, learning objectives, R scripts, and reading materials. As learners move through these modules, simple “step” scripts facilitate the construction of a complete RNA-seq analysis pipeline (Fig. 1). Currently, eight step scripts are provided, which include code for data preprocessing (steps 1 and 2), data visualization (step 3), accessing public data (step 4), carrying out differential gene expression (DGE) analysis (steps 5 and 6), using functional enrichment methods such as gene ontology (GO) and gene set enrichment analysis (GSEA) (step 7), and bundling code and outputs into dynamic Rmarkdown documents for transparency and reproducibility (step 8). This approach provides an opportunity to introduce statistical concepts in the context of real challenges that commonly arise during data analyses. For example, the module on data exploration introduces learners to experimental design considerations and to multivariate statistics and dimensional reduction as critical methods for identifying biological and technical sources of variance. Similarly, multiple-testing correction, linear models, and Bayesian inference become key concepts in the differential gene expression module. The course website contains additional reading material and supplemental videos for learners who want to explore these and other statistical concepts in more depth or for instructors who wish to dedicate additional lecture time to statistics.

At numerous points in this workflow, learners develop publication-quality graphics, opening the door to conversations about design aesthetics and crafting a narrative with genomic data. The course concludes by bundling all steps into an Rmarkdown document, providing an important context to discuss transparency and reproducibility in bioinformatics. To further emphasize the latter point, one module is dedicated to instruction on how to archive projects using GitHub and how to incorporate code into custom functions and R packages for reuse. Since all steps use the R programming language, learners build confidence and skills in coding as they progress through the course.

The modular structure of the course and stepwise nature of the coding not only accommodate learners with no prior experience in either RNA-seq or coding but also make it easy for instructors to modify the course content to include additional modules on statistics, related data types such as assay for transposase-accessible chromatin (ATAC-seq), or emerging technologies such as spatial transcriptomics. The next iteration of this course, for example, will include lectures and labs that explore single-cell RNA sequencing data from parasites and pathogen-infected host cells, to explore concepts around parasite development and host-pathogen interactions, respectively. Finally, many aspects of the course are generalizable well beyond transcriptomics data, and it would be feasible to adapt the course to focus on different ‘omic data types, including but not limited to microbiome profiling.

### Lessons learned from the COVID-19 pandemic.

The coronavirus disease 2019 (COVID-19) pandemic had a dramatic and abrupt impact on in-person instruction at schools around the world and underscored a desperate need for high-quality, free, online educational content for biomedical trainees. To help meet this need, we modified our course to be run virtually for a full semester starting in April 2020. Although the course had a strong online presence since its inception in 2015, the pandemic accelerated a move to make the course completely virtual. Several advantages of this move became immediately apparent. First, in-person bioinformatics courses often require specialized “active learning” media classrooms that offer numerous power outlets for laptop computers, round tables for group work, and multiple display screens for improved visibility. Such classrooms are difficult to find and are limited in seating. In contrast, shifting our course online allowed us to double the class size from about 60 students to 120 students. Teaching assistants that once perused the classroom now monitored a class message board (Slack) for student questions and held virtual recitations via video conferencing software (Zoom). Similarly, labs were run via video conferencing using “breakout room” features to randomly split the ∼120 students into small groups of 3 to 5 students. Teaching assistants and the instructor then circulated through each virtual breakout room to field questions and assist learners to overcome impediments. The virtual format also offered maximum flexibility during a time of great stress for learners. Furthermore, when offered in person, some learners struggled to keep up with modules that involved a mix of active coding and lecture. In contrast, virtual instruction with prerecorded video lectures made it simple to pause videos while coding. We also found that learners benefited from speed controls enabled on all videos, thus making it easy to move more quickly through familiar material while slowing down in more challenging areas.

**FIG 2:**
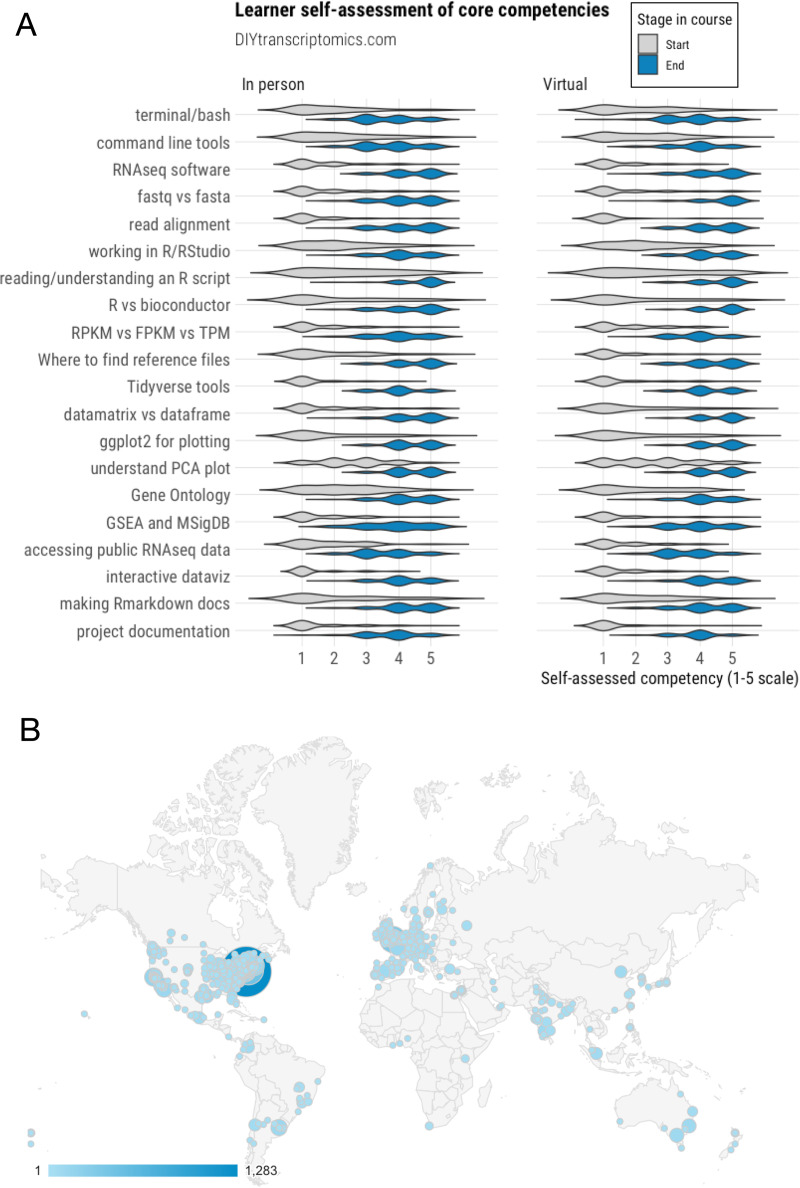
Learners show significant acquisition of skills regardless of whether material is delivered in person or via remote learning. (A) Self-reported data from 131 UPenn students who took the course either in person (*n* = 66) or virtually during the COVID-19 pandemic (*n* = 65). Students were asked to rank their competency in each area on a scale of 1 to 5, where 1 indicates “absolutely not” confident in a skill and 5 indicates “very confident.” RPKM, reads per kilobase per million; FPKM, fragments per kilobase per million; TPM, transcripts per million; PCA, principal-component analysis. (B) Google Analytics report showing the global distribution of over 17,000 users of the DIYtranscriptomics site since 1 January 2020. The size of the circles reflects site visits. The number of visitors per city is represented by blue shading of circles and is shown in the color key.

To empirically test whether the transition from in-person to virtual instruction had a detrimental impact on the acquisition of skills by learners, we compared results from a 20-question skill self-assessment survey completed by 66 in-person learners from 2019 with responses from 65 virtual learners from 2020 (*n* = 131 learners total) (Fig. 2A). Prior to starting the course, both in-person and virtual learners reported low confidence in their understanding of RNA-seq data, using command-line tools, the R programming language, and general aspects of data science and reproducible coding. After 15 modules, all students reported significant increases in all areas measured, regardless of whether they received instruction in person or virtually, demonstrating that the virtual format did not adversely impact the overall acquisition of skills by learners. Furthermore, the move to virtual instruction opened the course to learners from around the world (Fig. 2B). Since January 2020, over 17,000 people have visited the site. Although the majority originate from IP addresses in and around Philadelphia, PA, where our university is based, there were many users accessing the site from across the United States, Europe, India, and South America. By hosting our lecture videos on Vimeo and collecting detailed analytics on interactions of users with videos, we found that lectures had been viewed over 33,000 times and watched to completion over 12,000 times by over 4,000 unique viewers worldwide.

### Curriculum in the post-COVID-19 era.

The apparent success of the virtual format for this course raises the question of what should be done in a post-COVID-19 era when schools resume in-person instruction. Should virtual content be maintained? If the course remains fully virtual, then how would in-person instruction be used, if at all? These are questions that we and other educators are now wrestling with. Switching back to in-person instruction at the expense of maintaining strong virtual content not only would exclude learners from outside our institution but also would make us vulnerable yet again to significant disruptions from future local, national, or global emergencies. In contrast, keeping the course fully virtual without an in-person component ignores both an opportunity and a responsibility to engage students at our institution. A blended learning model that brings both concepts together offers an appealing solution. In this model, learners at our institution or elsewhere can watch the videos and learn asynchronously rather than attending traditional synchronous lectures in a classroom, while in-person classes focus on the data-driven labs described above (so-called “flipped classroom”). Lab content will still be made available online, raising the intriguing possibility of labs that mix in-person with virtual learners in small groups. Depending on course credit load and classroom size, instructors could opt to require learners to attend only a portion of the labs in person. Finally, an alternative model, and one that has spontaneously developed with this course at our institution, relates to the notion of “nanocourses” (15). Nanocourses are a short-course format that typically involves small groups of learners (e.g., a peer group from the same graduate program or all members of a particular laboratory) taking only a portion of the course, usually totaling about 5 to 6 h of instruction. The modular structure of our course readily accommodates a nanocourse model, where learners could spend approximately 6 h covering two modules and a lab.

### Extensible curriculum that can be adopted by LMICs.

An unexpected outcome of the nanocourse format described above is that students have found it relatively straightforward to use their own domain-specific data sets from neurobiology, cell biology, and model organisms such as *Drosophila* and Caenorhabditis elegans. This highlights that although currently focused on infectious diseases, the course can be easily extended to other areas of science, particularly since RNA-seq data are commonplace across biomedical research. This extensibility proves particularly useful when trying to engage students in the ever-changing landscape of infectious diseases since new (and newsworthy) outbreaks in human or veterinary medicine can easily be used as the basis for developing new data-driven labs. Of great concern is how this type of course can be successfully deployed to researchers in lower- and middle-income countries (LMICs) where endemic and emerging infectious diseases are major causes of childhood morbidity and mortality. To address this, the entire DIYtranscriptomics course, even the website itself, is available as a single GitHub repository (https://github.com/DIYtranscriptomics/DIYtranscriptomics.github.io), making it easy for any instructor to clone the course, modify the code, and quickly host their own version of the course with little effort. There remains the challenge of what to do when students do not have access to laptops with sufficient computing resources to install or run the course software. One appealing solution is the availability of containerized software and cloud computing infrastructure. For example, we have used CodeOcean to bundle all the course code and data into a reproducible cloud computing environment that requires only a Web browser and Internet access to run (16). Finally, in many areas of LMICs, a reliable Internet connection is not available. In these cases, videos can be freely downloaded for offline viewing, and the course GitHub repository can be cloned and used to run a local version of the website. Together, these resources provide multiple options for learners in LMICs to access rich bioinformatics content for infectious diseases.
